# Increased Expression of TIGIT/CD57 in Peripheral Blood/Bone Marrow NK Cells in Patients with Chronic Myeloid Leukemia

**DOI:** 10.1155/2020/9531549

**Published:** 2020-10-13

**Authors:** Danlin Yao, Ling Xu, Lian Liu, Xiangbo Zeng, Juan Zhong, Jing Lai, Runhui Zheng, Zhenyi Jin, Shaohua Chen, Xianfeng Zha, Xin Huang, Yuhong Lu

**Affiliations:** ^1^Department of Hematology, First Affiliated Hospital, Key Laboratory for Regenerative Medicine of Ministry of Education, Institute of Hematology, School of Medicine, Jinan University, Guangzhou, China; ^2^The Clinical Medicine Postdoctoral Research Station, Jinan University, Guangzhou, China; ^3^Department of Hematology, First Affiliated Hospital, Guangzhou Medical University, China; ^4^Department of Clinical Laboratory, First Affiliated Hospital, Jinan University, Guangzhou, China; ^5^Department of Hematology, Guangdong General Hospital (Guangdong Academy of Medical Sciences), Guangzhou, China

## Abstract

The antitumor activity of NK cells in patients with chronic myeloid leukemia (CML) is inhibited by the leukemia microenvironment. Recent studies have identified that the expression of TIGIT, CD57, and KLRG1 is related to the function, maturation, and antitumor capabilities of NK cells. However, the characteristics of the expression of these genes in the peripheral blood (PB) and bone marrow (BM) from patients with CML remain unknown. In this study, we used multicolor flow cytometry to assay the quantity and phenotypic changes of NK cells in PB and BM from de novo CML (DN-CML) and CML patients acquiring molecular response (MR-CML). We found that the expression of TIGIT, which inhibits NK cell function, is increased on CD56^+^ and CD56^dim^ NK cells in DN-CML PB compared with those in healthy individuals (HIs), and it is restored to normal in patients who achieve MR. We also found that the expression of CD57 on NK cells was approximately the same level in PB and BM from DN-CML patients, while decreased CD57 expression was found on CD56^+^ and CD56^dim^ NK cells in HI BM compared with PB. Additionally, those two subsets were significantly increased in DN-CML BM compared to HI BM. The expression of CD57 correlates with replicative senescence and maturity for human NK cells; therefore, the increase in TIGIT on PB NK cells together with an increase in CD57 on BM NK cells may explain the subdued NK cell antileukemia capacity and proliferative ability in DN-CML patients. These results indicate that reversing the immune suppression of PB NK cells by blocking TIGIT while improving the proliferation of BM NK cells via targeting CD57 may be more effective in removing tumor cells.

## 1. Introduction

Chronic myeloid leukemia (CML) is characterized by the expression of the *BCR/ABL1* fusion gene and the presence of the Philadelphia chromosome (Ph). The product of this fusion gene is a protein with deregulated tyrosine kinase activity, resulting in a malignant clonal disorder of the hematopoietic stem cells in the bone marrow (BM) and the accumulation of immature myeloid cells in peripheral blood (PB) [1]. The use of tyrosine kinase inhibitors (TKIs) leads to a complete remission rate reaching 83%; however, mutation in the ABL kinase domain results in certain treatment failure. Furthermore, long-lasting side effects of treatment and the cost of TKIs remain a problem [2, 3]. Therefore, the development of new TKI agents and combination therapies is urgently needed for CML patients [4].

There is an abundance of evidence that NK cells can exhibit potent antitumor activity against CML, acute myeloid leukemia (AML), and myelodysplastic syndromes (MDS). However, disease-associated mechanisms often inhibit the proper functions of endogenous NK cells, leading to inadequate tumor control and risk for disease progression [5–8]. As it is well known, the function of NK cells is precisely regulated by inhibitory and activating receptors. Higher surface expression of inhibitory receptors, such as natural killer group 2A (NKG2A), and lower expression of activating receptors, including natural killer group 2d (NKG2D) and DNAX accessory molecular-1 (DNAM-1), on cytotoxic NK cells were found in CML patients at diagnosis [9]. Recently, T cell immunoreceptor with immunoglobulin and ITIM domain (TIGIT) has been identified as a novel NK inhibitory receptor that can lead to NK cell exhaustion and dysfunction [10]. Inhibiting TIGIT on NK cells can restore the function of NK cells [11]. However, the expression of TIGIT on NK cells in de novo CML (DN-CML) patients remains unclear.

Human CD56^+^ NK cells represent 5-20% of peripheral blood mononuclear cells. According to the expression density of CD56 and CD16, these cells can be further subdivided into two subsets: CD56^bright^ NK cells, which represent a less mature population that is in charge of the production of cytokines, such as INF-*γ*, TNF-*α*, and MIP-1*α*, and CD56^dim^CD16^+^ NK cells (hereafter termed “CD56^dim^ NK”), which represent a more mature population responsible for cytotoxicity [12–14]. In addition to CD56, CD57 and killer cell lectin-like receptor subfamily G, member 1 (KLRG1) have been reported to be related to the terminal maturation state and homeostasis of NK cells, which can enhance their cytolytic ability [15–17]. CML patients who have achieved a major molecular response (MMR, BCR/ABL ≤ 0.1%) or molecular response^4.5^ (MR^4.5^, BCR/ABL ≤ 0.0032%) show a larger proportion of mature, cytolytic CD57^+^CD62L^−^ NK cells in PB with repertoires of activating and inhibitory receptors on NK cells restored to expression levels found in HIs [8]. However, whether CD57 and KLRG1 are altered in the PB and BM of DN-CML patients remains unknown.

Based on the importance of NK cells in antitumor function in CML, in this study, we assayed the TIGIT, CD57, and KLRG1 expression frequencies on NK cells and NK cell subsets in the PB and BM of DN-CML patients and patients who achieved a molecular response (MR-CML) after TKI treatment.

## 2. Material and Method

### 2.1. Samples

PB samples were collected from 13 de novo CML patients (DN-CML), 18 CML patients who achieved molecular response (MR), and 15 healthy individuals (HIs). The MR group included 3 different degrees of molecular response according to BCR/ABL1 level (>0.1%, before achievement of major molecular response, preMMR, *n* = 8, BCR‐ABL1 ≤ 0.1%; major molecular response, MMR, *n* = 8, BCR‐ABL1 ≤ 0.0032%; molecular response^4.5^, *n* = 2) [18]. BM were obtained by aspiration from de novo CML (*n* = 7), healthy individuals (*n* = 5) from hematopoietic stem cell transplant donors, and 3 case iron-deficiency anemia (IDA) as controls. Patients who had current or recent acute infection and those with autoimmune disease or diabetes mellitus were excluded. Clinical details of the patients are presented in Table 1. All sample collection was obtained with informed content from the patients and healthy volunteers. All procedures were conducted according to the guidelines of the Medical Ethics Committees of the Health Bureau of the Guangdong Province in China, and ethical approval was obtained from the Ethics Committee of the Medical School of Jinan University (No. (2016) Ethics Approval No. 010).

### 2.2. Immunophenotyping Analysis by Flow Cytometry

A total of 150 *μ*l of fresh whole blood or bone marrow was stained with CD45-BUV395 (clone: HI30), CD3-AF700 (clone: UCHT1), CD14-BV605 (clone: M5E2), CD19-BV605 (clone: SJ25C1), CD56-PE-CF594 (clone: B159), CD16-percp-cy5.5 (clone: 3G8), CD57-APC (clone: NK-1), TIGIT-BV421 (clone: A15153G), or KLRG1 (clone: SA231A2). All antibodies were used according to the manufacturer's instructions. Twenty microliters of absolute count microspheres (Thermo; Cat: C36950) was added to the samples for absolute cell number analysis. Samples were analyzed with a BD Verse flow cytometer (BD Biosciences, USA), and data analysis was performed with FlowJo software.

### 2.3. Statistical Analysis

Statistical analysis of unpaired samples was performed with Prism (GraphPad) using the independent-sample Wilcoxon test between two groups. *p* values of paired samples were calculated with Prism (GraphPad) using the Wilcoxon matched-pairs signed rank test. All data are represented as medians. Significance levels were defined as ns (not significant, *p* > 0.05). Values of *p* < 0.05 were considered significant.

## 3. Results and Discussion

### 3.1. TIGIT Is Increased on PB NK Subsets of DN-CML Patients

**Figure 1:**
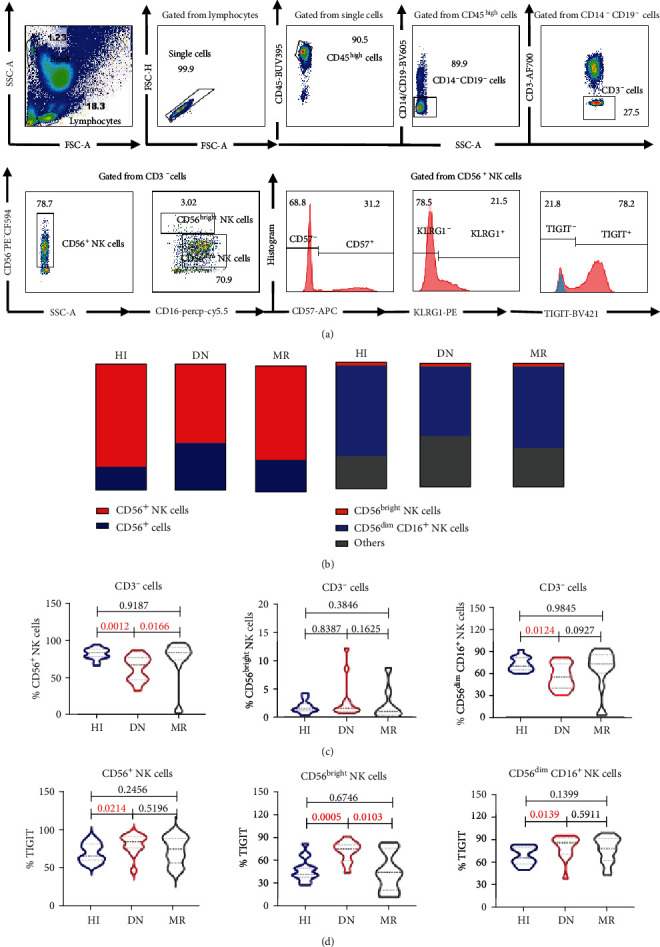
Increased levels of TIGIT^+^, TIGTI^+^CD56^bright^, and TIGIT^+^CD56^dim^CD16^+^ NK cells in DN-CML patients compared with MR patients and HIs. (a) The gating strategy for CD56^+^, CD56^bright^, and CD56^dim^ NK cells and the frequency of TIGIT, CD16, KLRG1, and CD57 on CD56^+^ NK cells are shown. Forward scatter area and height (FSC-H) are used to discriminate single cells. CD45 is used to discriminate the mature white blood cells. Monocytes and B cells are excluded using CD14 and CD19, and T cells are excluded using CD3. CD45^high^CD14^−^CD19^−^CD3^−^ population expressing CD56^+^, CD56^high^CD16^+^, and CD56^dim^CD16^+^ is gated as CD56^+^ NK cells, CD56^bright^ NK cells, and CD56^dim^ NK cells, respectively, and then, the expression of CD57, TIGIT, and KLRG1 on those NK subsets are analyzed [25]. (b) Summary of the altered distribution of CD56^+^ and CD56^−^ NK cells within the CD3^−^ population (left) and CD3^−^CD56^bright^ and CD56^dim^CD16^+^ NK cells as well as other cells within the CD3^−^ population (right) in PB from HIs (*n* = 15) and DN-CML (*n* = 13) and MR (*n* = 18) patients. (c) Frequency of CD56^+^, CD56^bright^, and CD56^dim^CD16^+^ NK cells in PB from HIs (*n* = 15) and DN-CML (*n* = 13) and MR (*n* = 18) patients. (d) Proportion of TIGIT^+^, TIGTI^+^CD56^bright^, and TIGIT^+^CD56^dim^CD16^+^ NK cells in PB from HIs (*n* = 15) and DN-CML (*n* = 13) and MR (*n* = 18) patients. All data are shown as medians ± quartiles. TIGIT: T cell immunoreceptor with Ig and ITIM domain; DN: de novo; CML: chronic myeloid leukemia; HIs: healthy individuals; MR: molecular response. The Mann–Whitney test was used for unpaired sample analysis, and *p* values < 0.05 were considered statistically significant.

To compare the proportion and the absolute number of NK subsets in different status of CML patients with those from HIs, we used nine antibodies for flow cytometry analysis. The gating strategy is shown in Figure 1(a). We found a decreased percentage (67%) of CD56^+^ NK cells accounting for the CD3^−^ population in PB from DN-CML patients compared with HIs (83.4%, *p* = 0.0012). The percentage of CD56^dim^ NK cells (55.05%) was also decreased in DN-CML patients compared with that in HIs (69.9%, *p* = 0.0124); however, the absolute NK cell numbers in the DN-CML patients were not different compared with those in HIs. These differences were restored to normal in MR-CML patients (Figures 1(b) and 1(c)). Previous studies have reported not only a significant decrease in the percentage but also the absolute number of NK cells in DN-CML patients [9, 19]. This difference may be due to differences in race and age, e.g., the ages of the DN-CML patients in our study were relatively young. In addition, the limited number of DN-CML patients in our study may also influence our findings. In addition to the number and percentage of NK cells that were changed in DN-CML patients, decreased NK cell function was also detected in DN-CML patients. For example, downregulation of NK cell-activating receptors CD161 and CD94/NKG2D and the natural cytotoxicity receptors NKp30 and NKp46 was also reported previously [8]. However, the expression of TIGIT, KLRG1, and CD57 and their association with the function, maturation, and antitumor ability of NK cells in DN-CML patients are unknown. Therefore, we evaluated the expression of the above markers on NK subsets from the PB of DN-CML and MR patients. We found that the expression level of TIGIT increased on total NK cells (84.25% *vs.* 65.8%, *p* = 0.0214) and the subsets CD56^bright^ (74.6% *vs.* 41.4%, *p* = 0.0005) and CD56^dim^ (83.70% *vs.* 65.30%, *p* = 0.0139) in DN-CML patients compared to that in HIs, while it was restored to normal in MR-CML patients (Figure 1(d)). However, the expression levels of KLRG1 and CD57 in the PB of DN-CML, MR-CML patients, and HIs had no differences (data not shown). Previous studies have found that TIGIT expression on tumor-infiltrating NK cells was associated with colon cancer progression and functional exhaustion of NK cells. In addition, in the setting of blocking TIGIT, NK cells not only exert a direct antitumor ability but also enhance CD8^+^ T cell function by increasing the secretion of INF-*γ*, TNF-*α*, and CD107a [10, 11]. CD56^bright^ NK cells mainly release cytokines (INF-*γ*, TNF-*α*) to assist in the antitumor ability of T cells, while CD56^dim^ NK cells directly kill tumors by cytotoxicity [20, 21]. Therefore, increased expression of TIGIT on NK subsets may be a reason for the NK cell dysfunction in DN-CML patients which is induced by continuous stimulation of leukemia antigens and blocking TIGIT may augment their antileukemia immune response. Further studies should evaluate the function of NK cells expressing high level of TIGIT in DN-CML patients and test the possibility of recovering NK cell function by blocking TIGIT.

### 3.2. CD57^+^ NK Cells Are Increased in the BM of DN-CML Patients

**Figure 2:**
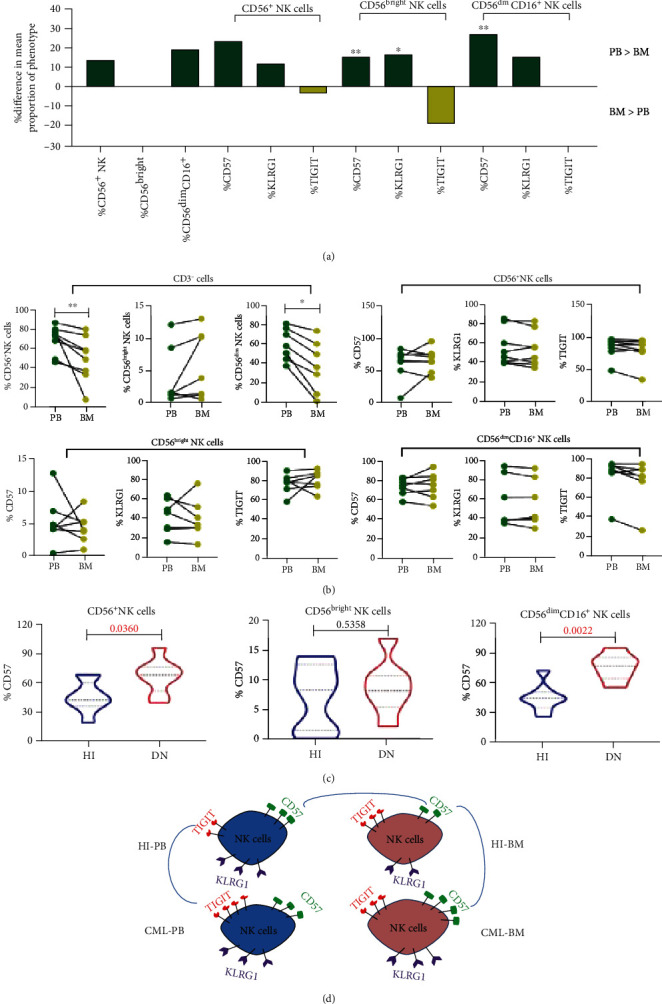
DN-CML BM NK cells had a significantly increased level of CD57 compared to NK cells in the BM from HIs. (a) Differences in the mean proportion of immunophenotypes in the BM from HIs (*n* = 8) and age-matched PB from HIs (*n* = 7; median age: 38; range: 21-51) are shown. The height of the bar signifies the amplitude of the difference between BM and PB samples (the PB median value is subtracted from the BM median value). The green bars above the axis represent immunophenotypes more prevalent in PB than BM samples. *p* values were computed using the nonparametric Mann–Whitney test between two groups. (b) The NK subset populations and expression of immunophenotypes in NK subsets from PB and matched BM from eight DN-CML patients. As the PB and BM were collected from the same patients, *p* values were computed using the parametric Mann–Whitney test between two groups. (c) Frequency of CD57^+^, CD57^+^CD56^bright^, and CD57^+^CD56^dim^ NK cells from HI BM (*n* = 8) and BM from 7 patients with DN-CML (*n* = 8). (d) Model illustrating the phenotypic differences of NK cells for BM and PB in HIs and BM and PB in DN-CML patients. All data are shown as medians ± quartiles. There is a significant difference between the two groups connected by the blue lines. Significance levels were defined as ns (not significant, *p* > 0.05). *p* values < 0.05 were indicated as significant.

NK cell development and functional maturation are complex and multistage processes that occur predominantly in the BM. Within the BM, the development of NK cell precursors and NK cells is mediated by a variety of cytokines and growth factors. Therefore, alterations in the BM microenvironment deeply impact the phenotype and function of NK cells. It is well known that the BM is the home niche for leukemia cells and it plays an important role in NK cell defense against tumors and viruses [22–24]. However, the number and phenotypic changes in the NK subsets in the BM of DN-CML patients remain unclear. In this study, we analyzed 7 paired PB and BM samples from DN-CML patients and found that a lower percentage of mature CD56^dim^ NK cells (36.9% *vs*. 73.90%, *p* = 0.0078) existed in the BM of DN-CML patients compared with PB, which was a pattern not different from what was found in HI BM and PB (52.23% *vs*. 71.32%, *p* = 0.032) (Figures 2(a) and 2(b)). Next, we compared the expression of TIGIT, CD57, and KLRG1 on NK subsets in the PB and BM of DN-CML patients and HIs. The results demonstrated that the CD57 expression level on the BM and PB NK cell subsets from DN-CML patients was approximately the same level (Figure 2(b)). However, the expression level of CD57 on the CD56^bright^ and CD56^dim^ NK cell subsets was significantly lower in HI BM compared with PB (44.51% *vs*. 71.87%, *p* = 0.0032; 25.34% *vs*. 52.46%, *p* = 0.0045, respectively) (Figure 2(a)). Thus, we found significantly increased CD57 expression on CD56^+^ and CD56^dim^ NK cells in DN-CML patient BM compared with HI BM (67.80% *vs*. 41.70%, *p* = 0.0360; 76.80% *vs*. 44.10%, *p* = 0.0022, respectively) (Figure 2(c)). As for the expression of KLRG1 and TIGIT, there were no differences between DN-CML patient PB and BM (Figure 2(b)). In addition, there were no differences between DN-CML patient BM and HI BM (data not shown). The above results indicated that BM NK cells from DN-CML patients have lost the normal phenotype existing in HI BM, i.e., low expression of CD57 and KLRG1, and increase in CD57 in particular could be a characteristic for BM NK cells in DN-CML patients. As reported by Lopez-Verges et al., CD57^+^CD56^dim^ NK cells are a terminally mature subset with a greater killer capacity, but their proliferation ability is defective [16]. Thus, we suspect that the BM microenvironment of DN-CML patients may stimulate the terminal maturation of BM NK cells to fight against leukemic cells at the cost of damaging their proliferation function.

## 4. Conclusion

We first described an increased level of TIGIT in PB NK cell subsets in DN-CML patients. We also found that NK cells from the BM of DN-CML patients tend to have a high expression of CD57. These results indicated that the increase in TIGIT on PB NK cells together with the increase in CD57 on BM NK cells may explain the subdued NK cell antileukemia capacity and proliferation ability in DN-CML patients. Thus, NK cells may be considered potential immunotherapy for DN-CML patients where blocking TIGIT on PB NK cells could reverse their immune suppression and targeting CD57 on BM NK cells could stimulate their proliferation in future treatment paradigms.

## Figures and Tables

**Table 1:** Sample characteristics.

Factor	HIs-PB	De novo CML-PB	MR-PB	HIs-BM	De novo CML-BM
The number of the case	15	13	18	8	7
Age (median; range) (years)	51 (21-82)	45.5 (32-74)	40 (21-79)	33 (17-51)	40 (32-82)
Gender (male/female)	9/6	9/4	8/10	3/5	4/3
BCR/ABL1 (IS) (%)	—	95.6 (13.4-240.0)	2.35 (0.005-9.1)	—	—
TKI duration (median, range) months	—	—	54 (1-108)	—	—

HIs: healthy individuals; CML: chronic myeloid leukemia; MR: molecular response; PB: peripheral blood; BM: bone marrow; IS: international standard; TKI: tyrosine kinase inhibitor.

## Data Availability

The datasets used and/or analyzed during the current study are available from the corresponding author upon reasonable request.

## Abbreviations

CML: Chronic myeloid leukemia
PB: Peripheral blood
BM: Bone marrow
DN-CML: De novo CML
MR-CML: CML patients acquiring molecular response
HIs: Healthy individuals
Ph: Philadelphia chromosome
TKIs: Tyrosine kinase inhibitors
AML: Acute myeloid leukemia
MDS: Myelodysplastic syndromes
NKG2A: Natural killer group 2A
NKG2D: Natural killer group 2d
DNAM-1: DNAX accessory molecular-1
TIGIT: T cell immunoreceptor with immunoglobulin and ITIM domain
KLRG1: Killer cell lectin-like receptor subfamily G, member 1
MMR: Major molecular response
MR^4.5^: Molecular response^4.5^
preMMR: Before achievement of major molecular response.

## References

[1] Faderl S., Talpaz M., Estrov Z., O'Brien S., Kurzrock R., Kantarjian H. M. (1999). The biology of chronic myeloid leukemia. *The New England Journal of Medicine*.

[2] O'Brien S. G., Guilhot F., Larson R. A. (2003). Imatinib compared with interferon and low-dose cytarabine for newly diagnosed chronic-phase chronic myeloid leukemia. *The New England Journal of Medicine*.

[3] Heaney N. B., Holyoake T. L. (2007). Therapeutic targets in chronic myeloid leukaemia. *Hematological Oncology*.

[4] Lee H. R., Baek K. H. (2019). Role of natural killer cells for immunotherapy in chronic myeloid leukemia (review). *Oncology Reports*.

[5] Carlsten M., Jaras M. (2019). Natural killer cells in myeloid malignancies: immune surveillance, NK cell dysfunction, and pharmacological opportunities to bolster the endogenous NK cells. *Frontiers in Immunology*.

[6] Pizzolo G., Trentin L., Vinante F. (1988). Natural killer cell function and lymphoid subpopulations in acute non-lymphoblastic leukaemia in complete remission. *British Journal of Cancer*.

[7] Kiladjian J. J., Bourgeois E., Lobe I. (2006). Cytolytic function and survival of natural killer cells are severely altered in myelodysplastic syndromes. *Leukemia*.

[8] Hughes A., Clarson J., Tang C. (2017). CML patients with deep molecular responses to TKI have restored immune effectors and decreased PD-1 and immune suppressors. *Blood*.

[9] Chang M. C., Cheng H. I., Hsu K. (2018). NKG2A down-regulation by dasatinib enhances natural killer cytotoxicity and accelerates effective treatment responses in patients with chronic myeloid leukemia. *Frontiers in Immunology*.

[10] Wang F., Hou H. Y., Wu S. J. (2015). TIGIT expression levels on human NK cells correlate with functional heterogeneity among healthy individuals. *European Journal of Immunology*.

[11] Zhang Q., Bi J., Zheng X. (2018). Blockade of the checkpoint receptor TIGIT prevents NK cell exhaustion and elicits potent anti-tumor immunity. *Nature Immunology*.

[12] Handgretinger R., Lang P., Andre M. C. (2016). Exploitation of natural killer cells for the treatment of acute leukemia. *Blood*.

[13] Gregoire C., Chasson L., Luci C. (2007). The trafficking of natural killer cells. *Immunological Reviews*.

[14] Michel T., Poli A., Cuapio A. (2016). Human CD56^bright^ NK cells: an update. *Journal of Immunology*.

[15] Ilander M., Olsson-Strömberg U., Schlums H. (2017). Increased proportion of mature NK cells is associated with successful imatinib discontinuation in chronic myeloid leukemia. *Leukemia*.

[16] Lopez-Verges S., Milush J. M., Pandey S. (2010). CD57 defines a functionally distinct population of mature NK cells in the human CD56^dim^CD16^+^ NK-cell subset. *Blood*.

[17] Huntington N. D., Tabarias H., Fairfax K. (2007). NK cell maturation and peripheral homeostasis is associated with KLRG1 up-regulation. *Journal of Immunology*.

[18] Mahon F. X., Etienne G. (2014). Deep molecular response in chronic myeloid leukemia: the new goal of therapy?. *Clinical Cancer Research*.

[19] Toubert A., Turhan A., Guerci-Bresler A., Dulphy N., Réa D. (2018). Lymphocytes NK : un rôle majeur dans le contrôle immunologique de la leucémie myéloïde chronique. *Medical Science*.

[20] Poli A., Michel T., Theresine M., Andres E., Hentges F., Zimmer J. (2009). CD56^bright^ natural killer (NK) cells: an important NK cell subset. *Immunology*.

[21] Chan A., Hong D. L., Atzberger A. (2007). CD56^bright^ human NK cells differentiate into CD56dim cells: role of contact with peripheral fibroblasts. *Journal of Immunology*.

[22] Grzywacz B., Moench L., McKenna D. (2019). Natural killer cell homing and persistence in the bone marrow after adoptive immunotherapy correlates with better leukemia control. *Journal of Immunotherapy*.

[23] Shafat M. S., Gnaneswaran B., Bowles K. M., Rushworth S. A. (2017). The bone marrow microenvironment - home of the leukemic blasts. *Blood Reviews*.

[24] Stabile H., Fionda C., Santoni A., Gismondi A. (2018). Impact of bone marrow-derived signals on NK cell development and functional maturation. *Cytokine & Growth Factor Reviews*.

[25] Costanzo M. C., Creegan M., Lal K. G., Eller M. A. (2015). OMIP-027: functional analysis of human natural killer cells. *Cytometry Part A*.

